# The effectiveness of antioxidant vitamins C and E in reducing myocardial infarct size in patients subjected to percutaneous coronary angioplasty (PREVEC Trial): study protocol for a pilot randomized double-blind controlled trial

**DOI:** 10.1186/1745-6215-15-192

**Published:** 2014-05-29

**Authors:** Ramón Rodrigo, Daniel Hasson, Juan C Prieto, Gastón Dussaillant, Cristóbal Ramos, Lucio León, Javier Gárate, Nicolás Valls, Juan G Gormaz

**Affiliations:** 1 Molecular and Clinical Pharmacology Program, Institute of Biomedical Sciences, Faculty of Medicine, University of Chile, Santiago, Chile; 2 Cardiovascular Department, University of Chile Clinical Hospital, Santiago, Chile; 3 Department of Radiology, University of Chile Clinical Hospital, Santiago, Chile; 4 Cardiovascular Center, San Borja Arriarán Clinical Hospital, Santiago, Chile

**Keywords:** Percutaneous coronary angioplasty, Ischemia-reperfusion, Oxidative stress, Vitamin C, Vitamin E, Acute myocardial infarction

## Abstract

**Background:**

Acute myocardial infarction (AMI) is the leading cause of mortality worldwide. Oxidative stress has been involved in the ischemia-reperfusion injury in AMI. It has been suggested that reperfusion accounts for up to 50% of the final size of a myocardial infarct, a part of the damage likely to be prevented.Therefore, we propose that antioxidant reinforcement through vitamins C and E supplementation should protect against the ischemia-reperfusion damage, thus decreasing infarct size.

The PREVEC Trial (Prevention of reperfusion damage associated with percutaneous coronary angioplasty following acute myocardial infarction) seeks to evaluate whether antioxidant vitamins C and E reduce infarct size in patients subjected to percutaneous coronary angioplasty after AMI.

**Methods/Design:**

This is a randomized, 1:1, double-blind, placebo-controlled clinical trial.

The study takes place at two centers in Chile: University of Chile Clinical Hospital and San Borja Arriarán Clinical Hospital.

The subjects will be 134 adults with acute myocardial infarction with indication for percutaneous coronary angioplasty.

This intervention is being performed as a pilot study, involving high-dose vitamin C infusion plus oral administration of vitamin E (Vitamin-treatment group) or placebo (Control group) during the angioplasty procedure. Afterward, the Vitamin-treatment group receives oral doses of vitamins C and E, and the Control group receives placebo for 84 days after coronary angioplasty.

Primary outcome is infarct size, assessed by cardiac magnetic resonance (CMR), measured 6 and 84 days after coronary angioplasty.

Secondary outcomes are ejection fraction, measured 6 and 84 days after coronary angioplasty with CMR, and biomarkers for oxidative stress, antioxidant status, heart damage, and inflammation, which will be measured at baseline, at the onset of reperfusion, 6 to 8 hours after revascularization, and at hospital discharge.

**Discussion:**

The ischemia-reperfusion event occurring during angioplasty is known to increase myocardial infarct size. The cardioprotective benefits of high doses of vitamin C combined with vitamin E have not been fully explored. The PREVEC Trial seeks to determine the suitability of the therapeutic use of vitamins C and E against the reperfusion damage produced during angioplasty.

Patient recruitment opened in February 2013. The trial is scheduled to end in March 2016.

**Trial registration:**

ISRCTN56034553

## Background

Primary percutaneous coronary angioplasty (PCA) is recognized as the most useful way to recover coronary flow in the context of acute myocardial infarction (AMI). Myocardial salvage and limitation of infarct-size expansion are the principal mechanisms whereby patients with ST-segment-elevation myocardial infarction (STEMI) benefit from reperfusion [1]. Despite these important advances in pharmacologic treatment and reperfusion strategies, heart failure is a functional consequence of AMI that determines poor long-term prognosis in coronary patients [2]. Among others, global left ventricular function has always been viewed as an important prognostic factor after AMI. Different strategies have been assayed to preserve left ventricular function and reduce infarct size in patients undergoing primary PCA for STEMI [3,4], but no clinical benefits have been obtained.

Currently, it is widely accepted that cardiac magnetic resonance (CMR) imaging is the gold-standard method to measure infarct size associated with AMI in clinical practice [5-8] and in clinical trials [9]. CMR can also measure accurately and reproducibly ejection fraction, ventricular volumes and cardiac mass [5]. Infarct size has been deemed an “important trial end-point” in the Joint ESC/ACCF/AHA/WHF Task Force for the Redefinition of Myocardial Infarction [10] and is a well-known outcome in trials that evaluate amelioration of reperfusion injury, [11-15] because its variation reflects the interaction of multiple physiologic and metabolic factors while providing a direct measure of the amount of myocardial cell loss [16]. This direct measure is especially valuable in the context of AMI, in which functional measures, due to the phenomena of myocardial stunning or myocardial hibernation, may not reflect the long-term compromise of the heart.

Reperfusion injury presents as damage to the myocardium after blood restoration subsequent to a critical period of coronary occlusion [17]. Ischemia–reperfusion is a clinical problem associated with procedures such as thrombolysis, angioplasty, and coronary bypass surgery, which are commonly used to establish the blood reflow and minimize the damage of the heart due to severe myocardial ischemia. Reperfusion injury includes a series of events: (a) reperfusion arrhythmias, (b) no-reflow phenomenon “microvascular damage, (c) myocardial stunning “reversible mechanical dysfunction,” and (d) lethal reperfusion “cell death,” which may occur either together or separately [18,19].

Two main hypotheses, oxidative stress and Ca^2+^ overload, have been proposed to explain the pathogenesis of ischemia–reperfusion injury [20,21]. Concerning this, oxidative stress, which is usually associated with increased formation of reactive oxygen species (ROS), modifies phospholipids and proteins, leading to lipid peroxidation and oxidation of thiol groups; these changes alter membrane permeability and configuration and generate functional modification of various cellular proteins [22].

Several studies have proposed the essential role of ROS in the pathogenesis of myocardial ischemia–reperfusion injury. In ischemic-reperfused hearts, many alterations, such as depression in contractile function, arrhythmias, change in gene expression, and loss of adrenergic pathways, have been observed [23]. Similar changes have been reported in hearts perfused with various ROS-generating systems. Furthermore, pretreatment of cardiac subcellular organelles with ROS showed similar changes. Thus, alterations in the myocardium during ischemia–reperfusion were suggested to be in part due to oxidative stress. In addition, ischemia–reperfusion was found to increase H_2_O_2_, cytosolic free Ca^2+^ concentration, malondialdehyde (MDA) content, and the formation of conjugated dienes in the heart. Treatment of the heart in animal models with antioxidant enzymes, superoxide dismutase (SOD), plus catalase protected against these changes [24,25]. ROS seem to increase significantly after a few minutes of reperfusion, but the increase during ischemia alone is still controversial.

On the basis of these changes, it has been suggested that the increase of superoxide anion and other ROS during reperfusion leads to lipid peroxidation and sulfhydryl group oxidation. It has been demonstrated that endothelial cells, inflammatory cells (that is, neutrophils and macrophages), and cardiomyocytes are all capable of generating ROS through several enzymatic reactions. It has been proposed that a burst of ROS from endothelial cells and cardiomyocytes during early reperfusion can influence nearby neutrophils, setting up a local cycle of amplified cellular response through released inflammatory mediators.

Furthermore, neutrophils become sensitized (primed) to activating factors, such as chemotactic cytokines, after they adhere to the endothelium, and thus generate much greater quantities of ROS. After the initial burst of ROS at the onset of reperfusion, later events such as transendothelial migration of neutrophils and macrophages, might participate in delayed ROS generation during reperfusion [26,27]. Activated neutrophils produce superoxide as a cytotoxic agent as part of the respiratory burst via the action of membrane-bound NADPH oxidase on molecular oxygen. Neutrophils also produce the free radical nitric oxide (NO) that can react with superoxide to produce peroxynitrite, the most powerful oxidant agent of nitrogen-reactive species (NOS), which may decompose to form hydroxyl radical [28].

In AMI, a clinical model of oxidative stress, ROS are generated in the ischemic myocardium, especially after reperfusion. ROS directly injure the cell membrane and cause cell death [29]. However, ROS also stimulate signal transduction to elaborate inflammatory cytokines(for example, tumor necrosis factor-α (TNF-α), interleukin (IL)-1β, and IL-6), in the ischemic region and surrounding myocardium as a host reaction. Inflammatory cytokines also regulate cell survival and cell death in the chain reaction with ROS. Apoptosis or programmed cell death is a distinct form of destruction of the cell, which is associated with synthesis of enzymes that degrade and fragment its own DNA. Updated information suggests that ischemia followed by reperfusion significantly induces myocardial injury by an apoptotic death pathway.

To understand the potential signaling mechanisms involved in ROS-triggered apoptosis, recent reports showed that cytosolic Ca^2+^overload and enhanced activity of the mitogen-activated protein kinase (MAPK) family during reperfusion can participate in induction of ROS-mediated apoptosis, in addition to necrosis, and eventually could be a determinant in infarct size [30].

Cell death was once viewed as unregulated. It is now clear that at least a portion of cell death is a regulated cell-suicide process. This type of death can exhibit multiple morphologies. One of these, apoptosis, has long been recognized to be actively mediated, and many of its underlying mechanisms have been elucidated. Moreover, necrosis, the traditional example of unregulated cell death, is also regulated in some instances. Autophagy is usually a survival mechanism but can occur in association with increased ROS, leading to cell death. Little is known, however, about how autophagic cells die [31]. Apoptosis, necrosis, and autophagy occur in cardiac myocytes during myocardial infarction, ischemia-reperfusion, and heart failure. Pharmacologic or genetic inhibition of apoptosis and necrosis lessens infarct size and improves cardiac function in these disorders [32].

ROS and NOS are major initiators of myocardial damage during reperfusion. Accordingly, AMI is usually initiated by myocardial ischemia due to coronary artery obstruction. In the ischemic myocardium, ROS are generated by a prooxidant state especially enhanced after reperfusion. Thus, neutrophils are the primary source of ROS during reperfusion. Endothelial cells and cardiomyocytes can also generate ROS. Increased ROS production is mainly due to activation of xanthine oxidase (enzyme that catalyzes the formation of uric acid with the co-production of superoxide) in endothelial cells [33], mitochondrial electron-transport chain reactions in cardiomyocytes, and NADPH oxidase in inflammatory cells [34]. Under these conditions, the enzymatic antioxidant effect is relevant against the detrimental effects of ROS. In agreement with this view, it has been reported that the transgenic mice in which superoxide dismutase (SOD) is overexpressed, infarct size is markedly reduced [35,36]. Accordingly, allopurinol, a xanthine oxidase inhibitor, has been demonstrated to block the superoxide production in ischemia–reperfusion settings involving organs such as heart [37], liver [33], kidney [38], and small intestine [39].

Therefore, it should be expected that a reinforcement of the antioxidant defense system through ROS scavengers results in a cardioprotective effect during the myocardial reperfusion. After an ischemic episode of the myocardium, left ventricle remodeling is known to occur; although its underlying mechanism is multifactorial, ROS and inflammatory cytokines may cause a cardiodepressive reaction [40-42]. It is of interest that ROS also stimulate the production of inflammatory cytokines and, inversely, inflammatory cytokines stimulate ROS formation. In the chronic stage, ROS and inflammatory cytokines activate the matrix metalloproteinases [43,44], thereby eliciting degradation of collagens, which may cause a slippage in myofibrillar alignment, causing left ventricular dilation [45].

Major evidence exists on the contribution of ROS to myocardial damage in AMI in humans. Therefore, it should be expected that treatments with antioxidant agents or upregulation of endogenous antioxidant enzymes could protect against reperfusion injury. Some studies have suggested that antioxidant agents attenuate the left ventricular remodeling after AMI. In patients with AMI subjected to primary percutaneous transluminal coronary angioplasty, the pretreatment with the inhibitor of xanthine oxidase allopurinol resulted in effective inhibition of the generation of oxygen-derived radicals during reperfusion therapy and the recovery of left ventricular function [46]. More recently, the administration of edaravone, a free radical scavenger, in patients with AMI just before reperfusion, reduced significantly the infarct size and reperfusion arrhythmia [47]. However, other attempts, such as an intravenous bolus of either superoxide dismutase [48] or trimetazidine [49], showed no beneficial effects in the outcome of patients.

Most investigations on the health-protective effects of vitamins C and E have been focused merely on their antioxidant power. Nevertheless, the biologic properties of α-tocopherol and ascorbic acid have overwhelmed their antioxidant effects. Despite the enormous interest in antioxidant vitamins as potential protective agents against the development of human disease, the real contributions of such compounds to health maintenance and the mechanisms whereby they act remain unclear. Antioxidants, as well as numerous cardioprotective strategies for reducing lethal reperfusion injury, have been administered in patients with AMI [50]. Although the scientific rationale, epidemiologic data, and retrospective studies have been persuasive, prospective, randomized, placebo-controlled trials have so far failed to verify the actual benefit of antioxidant vitamins in human diseases [51-54]. Among the possible contributory factors likely to account for this discrepancy,the lack of consideration of basic aspects, such as the pharmacokinetic properties of antioxidant vitamins, will be discussed later. In agreement with this view, previous attempts to reduce free radical production after PCA for AMI by oral administration of vitamin C, failed to attenuate the increased production of F2-isoprostanes [55]. In turn, Jaxa-Chamiec *et al*. [56], performing a randomized, double-blind, placebo-controlled multicentric study in 800 patients, analyzed the effects of combined vitamins C and E, through infusion and capsules, could not demonstrate a major effect of this antioxidant treatment on the clinical outcome of patients, although diabetes patients showed a reduction in 30-day cardiac mortality [57]. It should be noted that the authors recognize as a limitation of the study the fact that the dose of vitamin C used in the study increases its plasma levels only up to 0.1 m*M*, a concentration 100 times lower than that required to scavenge superoxide anion.

Vitamin E, mainly α-tocopherol, is the major peroxyl radical scavenger in biologic lipid phases such as membranes or LDL [58,59]. The antioxidant action has been ascribed to its ability to act chemically as a lipid-based free radical chain-breaking molecule, thereby inhibiting lipid peroxidation through its own conversion into an oxidized product, α-tocopheroxyl. α-Tocopherol can be restored by reduction of the α-tocopheroxyl radical with redox-active reagents like vitamin C or ubiquinol [60]. In clinical studies of ischemia-reperfusion injury, positive effects of a multivitamin antioxidant solution, including vitamin E, were described for revascularization of the lower extremities, kidney transplantation, liver surgery, and aortic aneurysm repair [61-64]. Preoperative administration of vitamin E is safe, and it may have beneficial effects by reducing the impact of ischemia-reperfusion injury in liver surgery [65]. However, homologous studies in AMI are still lacking.

Regarding vitamin C, intraarterial administration of high doses of ascorbate has been demonstrated to abolish the *in vivo* effects of superoxide anion in the impairment of vascular endothelial function in subjects with essential hypertension [66]. In addition, recent *in vitro* studies have also been successful to study the effects of oxidative stress with and without this vitamin C concentration, thus validating the use of this concentration of ascorbate to counteract the effects of oxidative stress [66]. Vitamin C concentration in plasma is tightly controlled, and excess of vitamin C is excreted as a function of dose, being completely saturated at doses of 400 mg daily and higher, producing a steady-state plasma concentration of approximately 80 μ*M*[67]. Unfortunately, this concentration is not enough to scavenge superoxide anion. Therefore, in settings accompanied by oxidative stress, such as the myocardial ischemia-reperfusion cycle, a beneficial effect of oral administration of vitamin C in the prevention of oxidative damage should not be expected; however, intravenous infusion could be considered with this purpose. Indeed, superoxide reacts with NO at a rate 10^5^-fold greater than the rate at which superoxide reacts with ascorbic acid [68]. As a consequence, 10 m*M* ascorbate is needed to support its competition with NO for superoxide. In patients undergoing elective PCA, impaired microcirculatory reperfusion is improved by vitamin C infusion, suggesting that oxidative stress is implicated in such a phenomenon [69]. Also, in patients subjected to thrombolysis after AMI, superoxide dismutase in the blood was found to be significantly reduced, whereas the activity of the oxidant enzyme, xanthine oxidase, and MDA levels were found to be significantly increased. However, oral supplementation of vitamin C to the postreperfusion patients restored these parameters back to normal or near-normal levels [70]. Although higher ascorbate doses would be needed to reach a better protective effect, its biologic properties, other than that of scavenging ROS, may have some beneficial effect. The major source of ROS is their enzymatic production via NADPH oxidase, an enzyme subjected to downregulation by vitamin C. In addition, vitamin C prevents the oxidation of tetrahydrobiopterin, a cofactor of NO synthase that is highly sensitive to oxidation. When tetrahydrobiopterin is oxidized, endothelial nitric oxide synthase (eNOS) activity becomes uncoupled, resulting in the production of superoxide instead of NO, thus enhancing the oxidative damage [71].

Ascorbic acid and α-tocopherol act as potent hydrophilic and lipophilic antioxidants, respectively [72]. They also act synergistically; in aqueous compartments, ascorbic acid can recycle α-tocopherol in membranes by reducing the α-tocopheroxyl radical back to α-tocopherol [73]. Consequently, in vitamin-E-supplemented rat hearts, α-tocopherol diminishes rapidly without the addition of vitamin C during reperfusion [74].

Finally, it is noteworthy that vitamin C could also abrogate the beneficial effects of ischemic preconditioning in animal models, a phenomenon induced by a series of brief sublethal episodes of ischemia and reperfusion before a potentially lethal episode of ischemia that renders the heart more resistant to myocardial infarction [75].

### Pilot study objectives

It is hypothesized that patients subjected to percutaneous coronary angioplasty to restore the coronary blood flow previously impaired by an acute myocardial infarction, while receiving a short-term infusion of high doses of vitamin C, plus oral doses of the recommended dose of vitamin E, will have a smaller infarct size, as well as an attenuation of the functional and biochemical damage occurring during the reperfusion afterthe sudden loss of blood supply, as compared with placebo-treated patients.

The objectives of this study are to determine in patients subjected to PCA after AMI:

i. the efficacy of vitamins C and E in reducing myocardial infarct size;

ii. the protective effects of vitamin C and E in cardiac function, assessed by measurement of ejection fraction;

iii. the levels of lipid peroxidation and protein carbonylation at baseline and immediately after successful reperfusion, 6 to 8 hours after vascular recanalization and at discharge;

iv. the levels of antioxidant potential at baseline and immediately after successful reperfusion, 6 to 8 hours after vascular recanalization, and at discharge; and

v. the correlation between oxidative stress-related biomarkers and both the infarct size and ejection fraction at 6 and 84 days after vascular recanalization.

## Methods/Design

### Design

**Figure 1:**
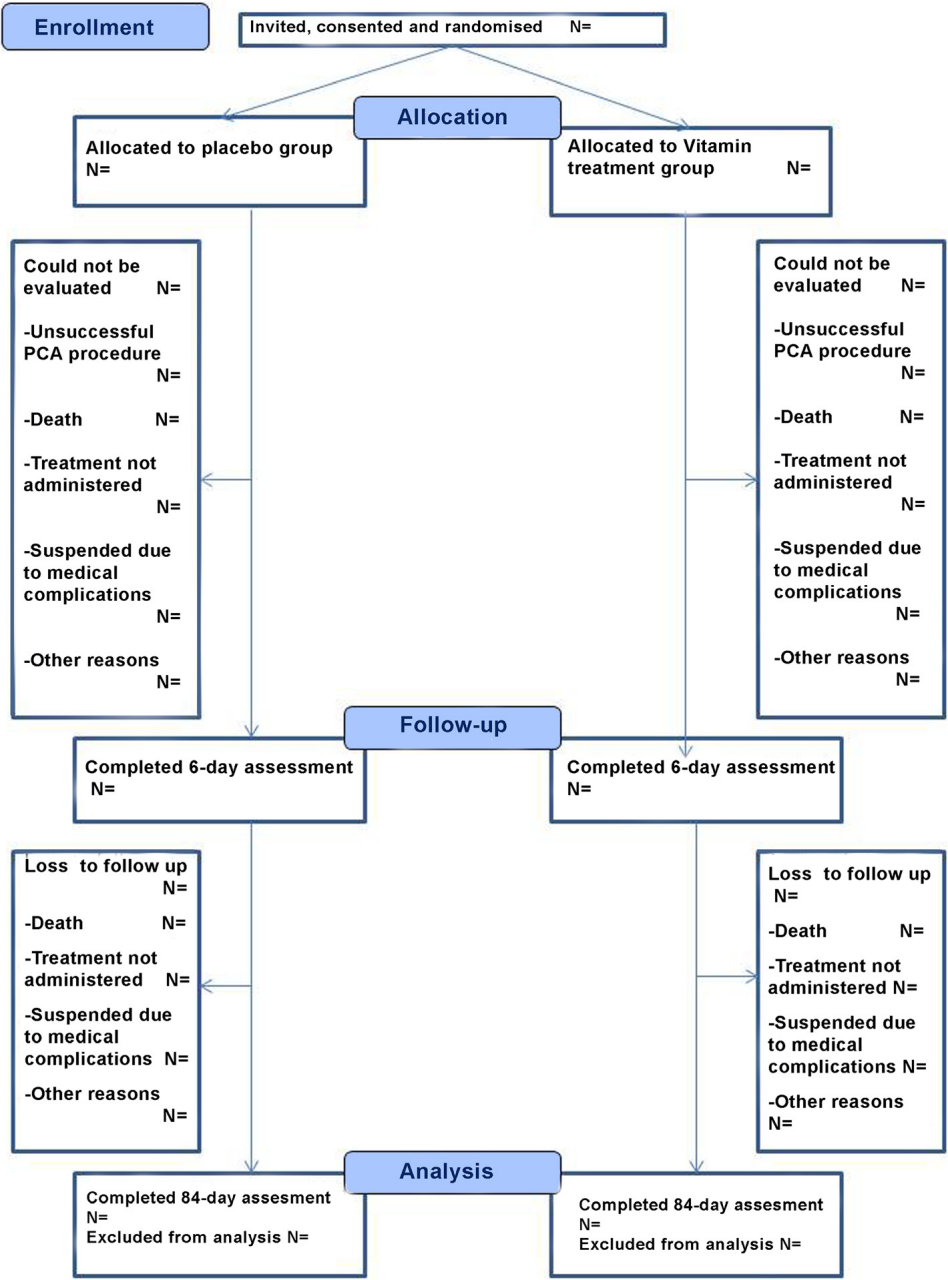
CONSORT flowchart for PREVEC Trial.

This double-blind, placebo-controlled, multicenter clinical trial will randomize in a 1:1 ratio to either placebo-treatment or vitamin-treatment groups. The study design is summarized in Figure 1.

### Study population

Patients of either sex, older than18 years, with an indication for primary percutaneous coronary angioplasty and experiencing their first acute myocardial infarction, admitted to University of Chile Clinical Hospital or San Borja Arriarán Clinical Hospital, will be invited to participate in the study. Inclusion and exclusion criteria are expressed in Tables 1 and 2, respectively.

**Table 1:** Inclusion criteria of the study protocol

**Inclusion criteria**	
1	Subjects may be of either sex and must be at least 18 years old
2	Subjects must have indication of primary percutaneous coronary angioplasty (PCA):
	-Angina or equivalent at least 120 minutes in duration
	- Electrocardiogram with ST-segment elevation myocardial infarction that concerns more than two contiguous leads (>2 mm)
3	Presentation within 12 hours of symptoms onset
4	First myocardial infarction
5	Primary PCA must show a pre-PCA TIMI flow of 0
6	Subject must be able and willing to sign informed consent

**Table 2:** Exclusion criteria of the study protocol

**Exclusion criteria**	
1	History of renal or hepatic insufficiency
2	History of renal lithiasis
3	History of heart failure (New York Heart Association III, IV)
4	Cardiogenic shock
5	Postprimary PCA TIMI grade flow of 0, 1, or 2
6	Any serious medical comorbidity that determines life expectancy <6 months
7	Current participation in any other clinical investigation
8	Pregnancy
9	Glucose 6-phosphate dehydrogenase deficiency

### Recruitment of patients

Patients will be screened by a member of the research team from those admitted to the emergency department of both clinical centers involved in the study. Patients diagnosed with AMI will be invited to participate in the study.

Informed consent will be obtained from suitable patients or their representative by one of the investigators or a delegated subinvestigator at each site. A member of the research team will answer all questions regarding the study and risks of the protocol procedure before the patient signs an informed-consent form.

### Sample size

Taking into account that until 50% infarct size is due to reperfusion damage, a minimal efficacy of the intervention is considered to reach a relative improvement up to 25% infarct size. Similar studies applying the same method have provided the accuracy of measurements as variance value. The sample size was calculated from the following formula [76]:

$$n=2{\left(\mathrm{ZS}+\mathrm{ZP}\right)}^{2}x\;S^{2}/{\left(\mathrm{MA}–\mathrm{MB}\right)}^{2}$$

Where *n* is the sample size for each group, “ZS” corresponds to the level of significance (5%), “ZP” represents the potency (80%), “MA” is the mean of supplemented group,MB, the mean of the placebo group (control), and S is the variance of CMR determination for assessment for IS. A 10% patient loss was considered for the purposes of this calculation. This calculation rendered a sample size of 66 patients for each branch (placebo and supplemented). This sample size is suitable to be covered by the population of patients being treated for PCA in the Cardiovascular Department, University of Chile Clinical Hospital, and Cardiovascular Center, San Borja Arriarán Clinical Hospital, during the estimated period (3 years).

### Randomization and followup

Patients will be randomly allocated to one of the two groups. The allocation sequence will be centrally generated, stored, and assigned by using online randomization software (http://www.randomization.com) in random permuted blocks, each consisting of a box containing six treatment kits allocated in a placebo-to-vitamin treatment, 1:1 ratio for each participating center.

Random allocation is generated by intervention of the technician responsible for the treatment assignment to recruited patients, according to the sequence already established through the randomization method indicated previously. The reasons for losses and exclusions after randomization will be provided.

Neither participants, nor care providers, nor the investigators are aware of the treatment assignments. Adverse events, unintended effects and technical problems will be recorded.

### Interventions

All patients will be asked to sign the informed consent before beginning the protocol. For those patients allocated to receive vitamin treatment, the intervention will be started as soon as the patient has signed the informed consent. The protocol will be started by administering an unique oral dose of vitamin E as α-tocopherol (800 IU) and an intravenous infusion of vitamin C as sodium ascorbate (320 m*M*) infused at a 10-ml/min flow rate during the initial hour and at 3 ml/min rate during the following 2 hours. Percutaneous coronary angioplasty will be performed about 30 minutes after initiating the vitamin C infusion. Oral doses of vitamin E (400 IU/day) and vitamin C (500 mg/12 hours) will be taken by the patients for 84 days after PCA.

For those patients allocated to receive placebo treatment, the 800-IU oral dose of vitamin E will be replaced for two vegetable oil capsules (400 mg each). The intravenous infusion of vitamin C will be replaced by an equal sodium chloride solution volume having the same osmolality as the vitamin C infusion administered to supplemented patients. The 400-IU oral doses of vitamin E will be replaced by vegetable oil (400 mg), and oral doses of vitamin C will be replaced with inert starch microgranules (500 mg). The pharmaceutical forms of vitamin C and E are indistinguishable from their respective placebos.

After the initial evaluation, patients will go to the catheterization laboratory. According to the standard protocol in AMI, an angiography will be performed to identify the infarct-related artery. The location and extent of AMI (number of arteries involved), percentage of stenosis, TIMI flow, type of stent, and so on, will be recorded. Immediately before coronary arteriography to confirm total occlusion of one coronary artery, a basal blood sample will be drawn from the antecubital vein. Three more blood samples will be obtained: immediately after successful reperfusion, at 6 to 8 hours after finishing the revascularization process and before hospital discharge.

Infarct size, the primary endpoint of the study, will be measured with cardiac magnetic resonance (CMR) at 6 and 84 days (12 weeks) after PCA. Both determination and data obtained by these procedures will be evaluated by two independent individuals with the purpose of applying the concordance kappa index. Adherence to oral intake of vitamins C and E or placebo will be evaluated by telephonic contact effectuated weekly by the nurse responsible for the trial patient´s care.

### Primary and secondary outcomes

Primary outcome will be infarct size, which will be measured with CMR at 6 and 84 days (12 weeks) after PCA. Both determination and data obtained by these procedures will be evaluated by two blinded independent individuals with the purpose of applying the concordance kappa index.

As our primary outcome is fully dependent on a high-quality blood-flow restoration to the ischemic myocardium region, we established as our criteria for successful reperfusion only patients who have a starting TIMI flow of 0 in the PCA and finish the procedure with TIMI flow 3, according to the most rigorous literature standards [77-79]. Secondary outcomes will be myocardial-damage biomarkers, oxidative stress- and inflammation-related biomarkers, and ejection fraction.

1. Ejection fraction: will be measured with cardiac magnetic resonance (CMR) at 6 and 84 days (12 weeks) after PCA. Both determination and data obtained by these procedures will be evaluated by two blinded independent individuals with the purpose of applying the concordance kappa index.

2. Oxidative stress-related biomarkers:

Plasma protein carbonylation

Antioxidant capacity of plasma: Ferric reducing ability of plasma (FRAP)

Plasma concentration of vitamins C and E

Thiol index: GSH/GSSG ratio in erythrocytes

Lipid peroxidation: F2-isoprostane and malondialdehyde levels in plasma and erythrocytes, respectively, will be measured.

3. Inflammation biomarkers:

High-sensitivity C-reactive protein

Leukocyte count by standard method

4. Myocardial-damage biomarkers:

Troponin, CK, and CK-MB will be measured in plasma with standard methods

Oxidative stress- and inflammation-related biomarkers and myocardial-damage biomarkers will be assessed through antecubital venous blood extraction, at the moment of enrollment (30 to 60 minutes before PCA), immediately after successful reperfusion, at 6 to 8 hours after finishing the revascularization process and before discharge, as previously described. The samples will be collected in chilled vacutainers containing disodium EDTA (final concentration, 4 m*M*) and centrifuged at 3,000 *g* for 10 minutes to separate the plasma from figurate elements. Erythrocytes will be subjected to hypotonic hemolysis by dilution with distilled water. Plasma and red blood cell lysates will be stored at -80°C until performing the biochemical analyses.

### Statistical analyses

Results of continuous variables will be expressed as mean ± standard deviation (SD). Comparison between parametric variables will be performed by using Student *t* test for unpaired samples. Nonparametric variables will be expressed as median (interquartile range) and compared through Wilcoxonrank-sum test. The significant differences for normally distributed variables will be compared with Student *t* test analysis of variance (ANOVA) for repeated measures. The significant differences for non-normally distributed variables will be compared by using the Mann–Whitney *U* test. Categoric variables will be expressed as numbers and frequencies (percentage). The Fisher Exact test with Katz approximation will be used to compare adverse-event frequencies.

Determination and data obtained for infarct size and ventricular function by cardiac resonance will be evaluated by two independent individuals with the purpose of applying the concordance kappa index. Among the limitations of using infarct size as an outcome, is that the use of final infarct size carries the risk of imbalances in baseline myocardium at risk, according to treatment group, as well as comparing different AMI locations, which may have different sensitivity to the therapy [80].

In addition to this, the groups may have variable time to reperfusion and other confounding variables that determine infarct size [81,82]. Whereas some of these difficulties may have been corrected through a careful stratification on entering the study, this option was discarded, given the small sample size of this study. We are currently considering an analysis of the data though a linear regression model, adjusted by all confounding variables, such as those mentioned before, and others present in the current literature [83]. *P*< 0.05 will be considered statistically significant. Results will be analyzed by using Stata version 8.0, Microsoft Excel,and Graphpad Prism 4.0.

### Ethical approval of the clinical trial

The research protocol was approved by the institutional ethics committees, including the University of Chile Faculty of Medicine (Approval certificate 060–2011; July 19, 2011), University of Chile Clinical Hospital (Approval certificate 53; July 27, 2011), San Borja Arriarán Clinical Hospital (Approval certificate 468–13; July 18, 2013), and also by the ethics committee of the National Fund for Scientific, Technological, and Innovation Development (FONDECYT) (Approval certificate G2-G3/590; May 24, 2012), the institution that approved the government grants for this clinical trial, all according to the Helsinki Declaration of the World Medical Association (2000).

### Trial management

Patients or their representatives will be asked to sign two copies of the informed consent. Patients will be given a copy of their consent form to keep for reference, and the other will be filed in the Investigator Site File on site. Data forms will be checked for completeness and merged into a master chart, which will be communicated to the study statistician. Patient confidentiality will be maintained at every stage.

Patients may withdraw from the trial or the trial treatment at any time without prejudice. Patients may be withdrawn from the study at the discretion of the local ethics committee for safety reasons. All adverse events and serious adverse events will be recorded during hospital stay and through patient communication while they are being subjected to ambulatory oral treatment. The study coordinator will conduct meetings with the study statistician on a regular basis, and the Chief Investigator will be made aware of all adverse events and serious adverse events as they happen.

## Discussion

Given the unpredictable nature of AMI, it is not feasible to obtain a basal myocardial image of patients; however, this trial also does not include performing an “acute” imaging on admission before the PCA (again, for feasibility reasons), but rather relies on performing a CMR on the sixth day after AMI. In this context, it is impossible to distinguish reperfusion damage from ischemic damage in the same patient, based in a single “6-day after” cardiac image. It would be expected on an imaging comparison among groups that on the 6^th^ and 84^th^ days, the placebo group exhibits, on average, a greater infarct size compared with the supplemented group. Any significant reduction of infarct size in the supplemented group could be attributed only to a decrease of reperfusion damage, because full coronary ischemia would not allow the arrival of ascorbate to the postoccluded artery segment. However, it will be impossible to determine whether a significant decrease of infarct size corresponds to total or partial reduction reperfusion damage.

To the best of our knowledge, no published reports have assessed human populations with high vitamin C levels in AMI patients during the PCA procedure; therefore, it is more difficult to compare data obtained with those in the current literature. In addition, given the characteristics of the study population, trial results are not applicable to patients having a second AMI, III or IV Killip score AMI, or other conditions established in the trial-exclusion criteria. Given that this study is being performed in a mostly Chilean population, its applicability to other ethnicities is uncertain.

Concerning the loss of data or patients during the study, patients undergoing a claustrophobic reaction will not be capable of being analyzed by CMR. Furthermore, due to the ambulatory nature of the trial, a small loss of patients may occur between the day 6 CMR and day 84 CMR. Given the multicentric nature of the study, the loss of samples is possible.

To our knowledge, no previous attempts have been made to use high doses of ascorbate to prevent or attenuate the myocardial damage caused by AMI. Pharmacokinetic studies on vitamin C in humans have shown that short-term infusion of high doses makes possible the reaching of peak concentrations even higher than 20 m*M*, but never being below 10 m*M* for at least 3 hours [84]. High doses of intravenous vitamin C have been reported to be remarkably safe, even when administered by infusions at a rate 3 times higher than that here proposed [85]. Nevertheless, patients having renal impairment or glucose 6-phosphate dehydrogenase deficiency, known possible complications of intravenous vitamin C, will be excluded from the present trial. However, a theoretic risk derived from high vitamin C levels is the development of kidney stones in unreported or first-time stone-forming patients [85]**.**

Regarding vitamin E possible adverse effects, current epidemiologic evidence (based on clinical trials that tested the effect of vitamin E supplementation in healthy participants and patients with various diseases) found that long-term supplementation with this nutrient shows a trend toward increasing slightly all-cause mortality (RR, 1.03; 95% CI, 1.00 to 1.05) independent of dose and exposition time [86]. However, the confidence interval is barely significant, because the lower interval is 1.00. In addition, as the Cochrane Meta-analysis explicitly excludes tertiary prevention trials, that is, randomized studies in which antioxidant supplements were used to treat a specific disease, such as trials involving patients with acute conditions (except nonmelanoma skin cancer), their conclusions are not applicable to our study.

Concerning the dosage, a previous meta-analysis reported that high-dosage supplementations (doses ≥400 IU/d) only increase all-cause mortality with expositions equal to or greater than 1 year [87]. Specifically in cardiovascular diseases, two recent meta-analysis that evaluate the efficacy of antioxidant supplements on these pathologies found no overall harmful effects in the analyzed endpoints [88,89], with the only exception of one controlled clinical trial [90]. However, those results are not consistent with the other related long-term large controlled clinical trials. In any case, that study supplemented the patients with twice our dose for more than a year, not being comparable in dose and timings with our study.

This novel strategy, by using innocuous and easily available substances, might significantly improve the clinical outcome of AMI patients, by reducing the infarct size, otherwise likely to result in working disability and diminution of both their life quality and expectancy.

Given the high incidence of AMI throughout the world and the innocuous and easily available substances used in the study, large-scale replication of this clinical trial worldwide seems feasible to the authors.

## Trial status

The PREVEC trial began recruitment in February 2013. Forty-three patients have been enrolled to date (3 June 2014) The trial is scheduled to end in March 2016.

## Abbreviations

AMI: Acute myocardial infarction; CMR: cardiac magnetic resonance; eNOS: endothelial nitric oxide synthase; IL-1β: interleukin-1β; IL-6: interleukin-6; MDA: malondialdehyde; MAPK: mitogen-activated protein kinase; NO: nitric oxide; PCA: percutaneous coronary angioplasty; ROS: reactive oxygen species; SOD: superoxide dismutase; STEMI: ST-segment elevation myocardial infarction; TNF-α: tumor necrosis factor-α.

## Competing interests

The authors report no conflicts of interest.

## Authors’ contributions

RR is the trial Chief Investigator. RR, JCP, GD, and CR contributed to the concept and study design and funding acquisition. JCP and JGG are responsible for patient recruitment and data collection in University of Chile Clinical Hospital. JG is responsible for patient recruitment and clinical data collection in San Borja Arriarán Clinical Hospital. GD is responsible for PCA intervention in the University of Chile Clinical Hospital. LL is responsible for PCA intervention in San Borja Arriarán Clinical Hospital. DH is responsible for oxidative-stress biomarker samples assessment. NV is responsible for the collection and interpretation of laboratory data. DH and RR drafted the first version of the manuscript. DH, JGG, and NV drafted the revised manuscript. All authors have commented on drafts of the article and have given final approval to this version.

## References

[B1] Braunwald E Myocardial reperfusion, limitation of infarct size, reduction of left ventricular dysfunction, and improved survival. should the paradigm be expanded? Circulation 1989 79 441 444 10.1161/01.CIR.79.2.441 2914356

[B2] van der Vleuten PA Rasoul S Huurnink W van der Horst IC Slart RH Reiffers S Dierckx RA Tio RA Ottervanger JP De Boer MJ Zijlstra F Effect of intravenous FX06 as an adjunct to primary percutaneous coronary intervention for acute ST-segment elevation myocardial infarction: results of the F.I.R.E. (Efficacy of FX06 in the Prevention of Myocardial Reperfusion Injury) trial BMC Cardiovasc Disord 2008 8 4 10.1186/1471-2261-8-4 18294397 PMC2278125

[B3] Atar D Petzelbauer P Schwitter J Huber K Rensing B Kasprzak JD Butter C Grip L Hansen PR Süselbeck T Clemmensen PM Marin-Galiano M Geudelin B Buser PT F.I.R.E. Investigators Effect of intravenous FX06 as an adjunct to primary percutaneous coronary intervention for acute ST-segment elevation myocardial infarction results of the F.I.R.E. (Efficacy of FX06 in the Prevention of Myocardial Reperfusion Injury) trial J Am Coll Cardiol 2009 53 720 729 10.1016/j.jacc.2008.12.017 19232907

[B4] Jang IK Pettigrew V Picard MH Kowey PR Demmel V Zile MR Tatsuno J Wackers FJ Hibberd M A randomized, double-blind, placebo-controlled study of the safety and efficacy of intravenous MCC-135 as an adjunct to primary percutaneous coronary intervention in patients with acute myocardial infarction: rationale and design of the evaluation of MCC-135 for left ventricular salvage in acute MI (EVOLVE) study J Thromb Thrombolysis 2005 20 147 153 10.1007/s11239-005-3267-4 16261287

[B5] von Knobelsdorff-Brenkenhoff F Schulz-Menger J Cardiovascular magnetic resonance imaging in ischemic heart disease J Magn Reson Imaging 2012 36 20 38 10.1002/jmri.23580 22696124

[B6] Perazzolo Marra M Lima JA Iliceto S MRI in acute myocardial infarction Eur Heart J 2011 32 284 293 10.1093/eurheartj/ehq409 21112897

[B7] Kim HW Farzaneh-Far A Kim R Cardiovascular magnetic resonance in patients with myocardial infarction: current and emerging applications J Am Coll Cardiol 2009 55 1 16 10.1016/j.jacc.2009.06.059 20117357

[B8] Martin TN Groenning BA Murray HM Steedman T Foster JE Elliot AT Dargie HJ Selvester RH Pahlm O Wagner GS ST-segment deviation analysis of the admission 12-lead electrocardiogram as an aid to early diagnosis of acute myocardial infarction with a cardiac magnetic resonance imaging gold standard J Am Coll Cardiol 2007 50 1021 1028 10.1016/j.jacc.2007.04.090 17825710

[B9] Desch S Eitel I de Waha S Fuernau G Lurz P Gutberlet M Schuler G Thiele H Cardiac magnetic resonance imaging parameters as surrogate endpoints in clinical trials of acute myocardial infarction Trials 2011 12 204 10.1186/1745-6215-12-204 21917147 PMC3182906

[B10] Thygesen K Alpert JS White HD Joint ESC/ACCF/AHA/WHF Task Force for the Redefinition of Myocardial Infarction J Am Coll Cardiol 2012 60 1581 1598 10.1016/j.jacc.2012.08.001 22958960

[B11] Faxon DP Gibbons RJ Chronos NA Gurbel PA Sheehan F The effect of blockade of the CD11/CD18 integrin receptor on infarct size in patients with acute myocardial infarction treated with direct angioplasty: the results of the HALT­MI study J Am Coll Cardiol 2002 40 1199 1204 10.1016/S0735-1097(02)02136-8 12383565

[B12] Zeymer U Suryapranata H Monassier JP Opolski G Davies J Rasmanis G Linssen G Tebbe U Schröder R Tiemann R Machnig T Neuhaus KL ESCAMI Investigators J Am Coll Cardiol 2001 38 1644 1650 10.1016/S0735-1097(01)01608-4 11704395

[B13] Baran KW Nguyen M McKendall GR Lambrew CT Dykstra G Palmeri ST Gibbons RJ Borzak S Sobel BE Gourlay SG Rundle AC Gibson CM Barron HV Limitation of Myocardial Infarction Following Thrombolysis in Acute Myocardial Infarction (LIMIT AMI) Study Group Circulation 2001 104 2778 2783 10.1161/hc4801.100236 11733394

[B14] Mahaffey KW Granger CB Nicolau JC Ruzyllo W Weaver WD Theroux P Hochman JS Filloon TG Mojcik CF Todaro TG Armstrong PW COMPLY Investigators Effect of pexelizumab, an anti-C5 complement antibody, as adjunctive therapy to fibrinolysis in acute myocardial infarction: the Complement inhibition in myocardial infarction treated with thromboLYtics (COMPLY) trial Circulation 2003 108 1176 1183 10.1161/01.CIR.0000087404.53661.F8 12925455

[B15] Ross AM Gibbons RJ Stone GW Kloner RA Alexander RW A Randomized, Double-Blinded, Placebo-Controlled Multicenter Trial of Adenosine as an Adjunct to Reperfusion in the Treatment of Acute Myocardial Infarction (AMISTAD-II) J Am Coll Cardiol 2005 45 1775 1780 10.1016/j.jacc.2005.02.061 15936605

[B16] Lowe JE Reimer KA Jennings RB Experimental infarct size as a function of the amount of myocardium at risk Am J Pathol 1978 90 363 379 623206 PMC2018154

[B17] Dhalla NS Elmoselhi AB Hata T Makino N Status of myocardial antioxidants in ischemia-reperfusion injury Cardiovasc Res 2000 47 446 456 10.1016/S0008-6363(00)00078-X 10963718

[B18] Bolli R Marbán E Molecular and cellular mechanisms of myocardial stunning Physiol Rev 1999 79 609 634 10221990 10.1152/physrev.1999.79.2.609

[B19] Murphy AM Heart failure, myocardial stunning, and troponin: a key regulator of the cardiac myofilament Congest Heart Fail 2006 12 32 38 16470090 10.1111/j.1527-5299.2006.04320.x

[B20] Ferrari R The role of mitochondria in ischemic heart disease J Cardiovasc Pharmacol 1996 28 1 10 8891865 10.1097/00005344-199600003-00002

[B21] Jahangiri A Leifert WR Kind KL McMurchie EJ Dietary fish oil alters cardiomyocyte Ca2+ dynamics and antioxidant status Free Radic Biol Med 2006 40 1592 1602 10.1016/j.freeradbiomed.2005.12.026 16632119

[B22] Hool LC Evidence for the regulation of L-type Ca2+ channels in the heart by reactive oxygen species: mechanism for mediating pathology Clin Exp Pharmacol Physiol 2008 35 229 234 18197892 10.1111/j.1440-1681.2007.04727.x

[B23] Rensing H Bauer I Kubulus D Wolf B Winning J Ziegeler S Bauer M Heme oxygenase-1 gene expression in pericentral hepatocytes through beta1-adrenoceptor stimulation Shock 2004 21 376 387 10.1097/00024382-200404000-00014 15179140

[B24] Dhalla NS Golfman L Takeda S Takeda N Nagano M Evidence for the role of oxidative stress in acute ischemic heart disease: a brief review M Can J Cardiol 1999 15 587 593 10350670

[B25] Xu Y Liu B Zweier JL He G Formation of hydrogen peroxide and reduction of peroxynitrite via dismutation of superoxide at reperfusion enhances myocardial blood flow and oxygen consumption in postischemic mouse heart J Pharmacol Exp Ther 2008 327 402 410 10.1124/jpet.108.142372 18685120 PMC2615247

[B26] Cooper D Stokes KY Tailor A Granger DN Oxidative stress promotes blood cell-endothelial cell interactions in the microcirculation Cardiovasc Toxicol 2002 2 165 180 10.1007/s12012-002-0002-7 12665663

[B27] Pavelková M Kubala L Cíz M Pavlík P Wagner R Slavík J Ondrásek J Cerný J Lojek A Blood phagocyte activation during open heart surgery with cardiopulmonary bypass Physiol Res 2006 55 165 173 15910174 10.33549/physiolres.930662

[B28] Hori M Nishida K Oxidative stress and left ventricular remodelling after myocardial infarction Cardiovasc Res 2009 81 457 464 19047340 10.1093/cvr/cvn335

[B29] van Dijk A Krijnen PA Vermond RA Pronk A Spreeuwenberg M Visser FC Berney R Paulus WJ Hack CE van Milligen FJ Niessen HW Inhibition of type 2A secretory phospholipase A2 reduces death of cardiomyocytes in acute myocardial infarction Apoptosis 2009 14 753 763 10.1007/s10495-009-0350-x 19421861

[B30] Matsui Y Takagi H Qu X Abdellatif M Sakoda H Asano T Levine B Sadoshima J Distinct roles of autophagy in the heart during ischemia and reperfusion: roles of AMP-activated protein kinase and Beclin 1 in mediating autophagy Circ Res 2007 100 914 922 10.1161/01.RES.0000261924.76669.36 17332429

[B31] Baines CP Kaiser RA Purcell NH Blair NS Osinska H Hambleton MA Brunskill EW Sayen MR Gottlieb RA Dorn GW Robbins J Molkentin JD Loss of cyclophilin D reveals a critical role for mitochondrial permeability transition in cell death Nature 2005 434 658 662 10.1038/nature03434 15800627

[B32] Juránek I Bezek S Controversy of free radical hypothesis: reactive oxygen species–cause or consequence of tissue injury? Gen Physiol Biophys 2005 24 263 278 16308423

[B33] Granger DN Role of xanthine oxidase and granulocytes in ischemia-reperfusion injury Am J Physiol 1988 255 1269 1275 10.1152/ajpheart.1988.255.6.H1269 3059826

[B34] Tan S Yokoyama Y Dickens E Cash TG Freeman BA Parks DA Xanthine oxidase activity in the circulation of rats following hemorrhagic shock Free Radic Biol Med 1993 15 407 414 10.1016/0891-5849(93)90040-2 8225022

[B35] Terada LS Dormish JJ Shanley PF Leff JA Anderson BO Repine JE Circulating xanthine oxidase mediates lung neutrophil sequestration after intestinal ischemia-reperfusion Am J Physiol 1992 263 394 401 10.1152/ajplung.1992.263.3.L394 1329531

[B36] Grisham MB Hernandez LA Granger DN Xanthine oxidase and neutrophil infiltration in intestinal ischemia Am J Physiol 1986 251 567 574 10.1152/ajpgi.1986.251.4.G567 3020994

[B37] Duilio C Ambrosio G Kuppusamy P DiPaula A Becker LC Zweier JL Neutrophils are primary source of O2 radicals during reperfusion after prolonged myocardial ischemia Am J Physiol Heart Circ Physiol 2001 280 2649 2657 10.1152/ajpheart.2001.280.6.H2649 11356621

[B38] Chen Z Siu B Ho YS Vincent R Chua CC Hamdy RC Chua BH Overexpression of MnSOD protects against myocardial ischemia/reperfusion injury in transgenic mice J Mol Cell Cardiol 1998 30 2281 2289 10.1006/jmcc.1998.0789 9925365

[B39] Wang P Chen H Qin H Sankarapandi S Becher MW Wong PC Zweier JL Overexpression of human copper, zinc-superoxide dismutase (SOD1) prevents postischemic injury Proc Natl Acad Sci U S A 1998 95 4556 4560 10.1073/pnas.95.8.4556 9539776 PMC22528

[B40] Frangogiannis NG Smith CW Entman ML The inflammatory response in myocardial infarction Cardiovasc Res 2002 53 31 47 10.1016/S0008-6363(01)00434-5 11744011

[B41] Ferdinandy P Danial H Ambrus I Rothery RA Schulz R Peroxynitrite is a major contributor to cytokine-induced myocardial contractile failure Circ Res 2000 87 241 247 10.1161/01.RES.87.3.241 10926876

[B42] Suematsu N Tsutsui H Wen J Kang D Ikeuchi M Ide T Hayashidani S Shiomi T Kubota T Hamasaki N Takeshita A Oxidative stress mediates tumor necrosis factor-alpha-induced mitochondrial DNA damage and dysfunction in cardiac myocytes Circulation 2003 107 1418 1423 10.1161/01.CIR.0000055318.09997.1F 12642364

[B43] Siwik DA Pagano PJ Colucci WS Oxidative stress regulates collagen synthesis and matrix metalloproteinase activity in cardiac fibroblasts Am J Physiol Cell Physiol 2001 280 53 60 10.1152/ajpcell.2001.280.1.C53 11121376

[B44] Deten A Hölzl A Leicht M Barth W Zimmer HG Changes in extracellular matrix and in transforming growth factor beta isoforms after coronary artery ligation in rats J Mol Cell Cardiol 2001 33 1191 1207 10.1006/jmcc.2001.1383 11444923

[B45] Rohde LE Ducharme A Arroyo LH Aikawa M Sukhova GH Lopez-Anaya A McClure KF Mitchell PG Libby P Lee RT Matrix metalloproteinase inhibition attenuates early left ventricular enlargement after experimental myocardial infarction in mice Circulation 1999 99 3063 3070 10.1161/01.CIR.99.23.3063 10368126

[B46] Guan W Osanai T Kamada T Hanada H Ishizaka H Onodera H Iwasa A Fujita N Kudo S Ohkubo T Okumura K Effect of allopurinol pretreatment on free radical generation after primary coronary angioplasty for acute myocardial infarction J Cardiovasc Pharmacol 2003 41 699 705 10.1097/00005344-200305000-00005 12717099

[B47] Tsujita K Shimomura H Kaikita K Kawano H Hokamaki J Nagayoshi Y Yamashita T Fukuda M Nakamura Y Sakamoto T Yoshimura M Ogawa H Long-term efficacy of edaravone in patients with acute myocardial infarction Circ J 2006 70 832 837 10.1253/circj.70.832 16799234

[B48] Flaherty JT Pitt B Gruber JW Heuser RR Rothbaum DA Burwell LR George BS Kereiakes DJ Deitchman D Gustafson N Recombinant human superoxide dismutase (h-SOD) fails to improve recovery of ventricular function in patients undergoing coronary angioplasty for acute myocardial infarction Circulation 1994 89 1982 1991 10.1161/01.CIR.89.5.1982 8181121

[B49] Downey JM Free radicals and their involvement during long-term myocardial ischemia and reperfusion Annu Rev Physiol 1990 52 487 504 10.1146/annurev.ph.52.030190.002415 2184765

[B50] Yellon DM Hausenloy DJ Myocardial reperfusion injury N Engl J Med 2007 357 1121 1135 10.1056/NEJMra071667 17855673

[B51] Lassnigg A Punz A Barker R Keznickl P Manhart N Roth E Hiesmayr M Influence of intravenous vitamin E supplementation in cardiac surgery on oxidative stress: a double-blinded, randomized, controlled study Br J Anaesth 2003 90 148 154 10.1093/bja/aeg042 12538369

[B52] Rodrigo R Guichard C Charles R Clinical pharmacology and therapeutic use of antioxidant vitamins Fund Clin Pharmacol 2007 21 111 127 10.1111/j.1472-8206.2006.00466.x 17391284

[B53] Sesso HD Buring JE Christen WG Kurth T Belanger C MacFadyen J Bubes V Manson JE Glynn RJ Gaziano JM Vitamins E and C in the prevention of cardiovascular disease in men: the Physicians' Health Study II randomized controlled trial JAMA 2008 300 2123 2133 10.1001/jama.2008.600 18997197 PMC2586922

[B54] Gaziano JM Glynn RJ Christen WG Kurth T Belanger C MacFadyen J Bubes V Manson JE Sesso HD Buring JE Vitamins E and C in the prevention of prostate and total cancer in men: the Physicians' Health Study II randomized controlled trial JAMA 2009 301 52 62 19066368 10.1001/jama.2008.862 PMC2774210

[B55] Guan W Osanai T Kamada T Ishizaka H Hanada H Okumura K Time course of free radical production after primary coronary angioplasty for acute myocardial infarction and the effect of vitamin C Jpn Circ J 1999 63 924 928 10.1253/jcj.63.924 10614835

[B56] Jaxa-Chamiec T Bednarz B Drozdowska D Gessek J Gniot J Janik K Kawka-Urbanek T Maciejewski P Ogórek M Szpajer M MIVIT Trial Group Antioxidant effects of combined vitamins C and E in acute myocardial infarction: the randomized, double-blind, placebo controlled, multicenter pilot Myocardial Infarction and VITamins (MIVIT) trial Kardiol Pol 2005 62 344 350 16059992

[B57] Jaxa-Chamiec T Bednarz B Herbaczynska-Cedro K Maciejewski P Ceremuzynski L MIVIT Trial Group Effects of vitamins C and E on the outcome after acute myocardial infarction in diabetics: a retrospective, hypothesis-generating analysis from the MIVIT study Cardiology 2009 112 219 223 10.1159/000151239 18698138

[B58] Upston JM Witting PK Brown AJ Stocker R Keaney JF Jr Effect of vitamin E on aortic lipid oxidation and intimal proliferation after arterial injury in cholesterol-fed rabbits Free Radic Biol Med 2001 31 1245 1253 10.1016/S0891-5849(01)00721-3 11705703

[B59] Terentis AC Thomas SR Burr JA Liebler DC Stocker R Vitamin E oxidation in human atherosclerotic lesions Circ Res 2002 90 333 339 10.1161/hh0302.104454 11861423

[B60] Shi H Noguchi N Niki E Comparative study on dynamics of antioxidative action of alpha-tocopheryl hydroquinone, ubiquinol, and alpha-tocopherol against lipid peroxidation Free Radic Biol Med 1999 27 334 346 10.1016/S0891-5849(99)00053-2 10468207

[B61] Rabl H Khoschsorur G Colombo T Petritsch P Rauchenwald M Költringer P Tatzber F Esterbauer H A multivitamin infusion prevents lipid peroxidation and improves transplantation performance Kidney Int 1993 43 912 917 10.1038/ki.1993.128 8479129

[B62] Cerwenka H Bacher H Werkgartner G El-Shabrawi A Quehenberger F Hauser H Mischinger HJ Antioxidant treatment during liver resection for alleviation of ischemia-reperfusion injury Hepatogastroenterology 1998 45 777 782 9684133

[B63] Wijnen MH Roumen RM Vader HL Goris RJ A multiantioxidant supplementation reduces damage from ischaemia reperfusion in patients after lower torso ischaemia: a randomised trial Eur J Vasc Endovasc Surg 2002 23 486 490 10.1053/ejvs.2002.1614 12093062

[B64] Rabl H Khoschsorur G Petek W Antioxidative vitamin treatment: effect on lipid peroxidation and limb swelling after revascularization operations World J Surg 1995 19 738 744 10.1007/BF00295919 7571673

[B65] Bartels M Biesalski HK Engelhart K Sendlhofer G Rehak P Nagel E Pilot study on the effect of parenteral vitamin E on ischemia and reperfusion induced liver injury: a double blind, randomized, placebo-controlled trial Clin Nutr 2004 23 1360 1370 10.1016/j.clnu.2004.05.003 15556258

[B66] Schneider MP Delles C Schmidt BM Oehmer S Schwarz TK Schmieder RE John S Superoxide scavenging effects of N-acetylcysteine and vitamin C in subjects with essential hypertension Am J Hypertens 2005 18 1111 1117 10.1016/j.amjhyper.2005.02.006 16109326

[B67] Graumlich JF Ludden TM Conry-Cantilena C Cantilena LR Jr Wang Y Levine M Pharmacokinetic model of ascorbic acid in healthy male volunteers during depletion and repletion Pharm Res 1997 14 1133 1139 10.1023/A:1012186203165 9327438

[B68] Jackson TS Xu A Vita JA Keaney JF Jr Ascorbate prevents the interaction of superoxide and nitric oxide only at very high physiological concentrations Circ Res 1998 83 916 922 10.1161/01.RES.83.9.916 9797340

[B69] Basili S Tanzilli G Mangieri E Raparelli V Di Santo S Pignatelli P Violi F Intravenous ascorbic acid infusion improves myocardial perfusion grade during elective percutaneous coronary intervention: relationship with oxidative stress markers JACC Cardiovasc Interv 2010 3 221 229 10.1016/j.jcin.2009.10.025 20170881

[B70] Bhakuni P Chandra M Misra MK Effect of ascorbic acid supplementation on certain oxidative stress parameters in the post reperfusion patients of myocardial infarction Mol Cell Biochem 2006 290 153 158 10.1007/s11010-006-9182-y 16718365

[B71] Virdis A Colucci R Fornai M Polini A Daghini E Duranti E Ghisu N Versari D Dardano A Blandizzi C Taddei S Del Tacca M Monzani F Inducible nitric oxide synthase is involved in endothelial dysfunction of mesenteric small arteries from hypothyroid rats Endocrinology 2009 150 1033 1042 10.1210/en.2008-1112 18927216

[B72] Niki E Noguchi N Tsuchihashi H Gotoh N Interaction among vitamin C, vitamin E, and beta-carotene Am J Clin Nutr 1995 62 1322S 1326S 7495227 10.1093/ajcn/62.6.1322S

[B73] Rodrigo R Libuy M Feliú F Hasson D Molecular basis of cardioprotective effect of antioxidant vitamins in myocardial infarction Biomed Res Int 2013 2013 437613 23936799 10.1155/2013/437613 PMC3726017

[B74] Molyneux CA Glyn MC Ward BJ Oxidative stress and cardiac microvascular structure in ischemia and reperfusion: the protective effect of antioxidant vitamins Microvasc Res 2002 64 265 277 10.1006/mvre.2002.2419 12204651

[B75] Tsovolas K Iliodromitis EK Andreadou I Zoga A Demopoulou M Iliodromitis KE Manolaki T Markantonis SL Kremastinos DT Acute administration of vitamin C abrogates protection from ischemic preconditioning in rabbits Pharmacol Res 2008 57 283 289 10.1016/j.phrs.2008.02.003 18353674

[B76] van Belle G Statistical Rules of Thumb 2nd ed 2008 Hoboken, NJ: Wiley 30

[B77] Brener SJ Maehara A Dizon JM Fahy M Witzenbichler B Parise H El-Omar M Dambrink JH Mehran R Oldroyd K Gibson CM Stone GW Relationship between myocardial reperfusion, infarct size, and mortality: the INFUSE-AMI (Intracoronary Abciximab and Aspiration Thrombectomy in Patients with Large Anterior Myocardial Infarction) trial JACC Cardiovasc Interv 2013 6 718 724 10.1016/j.jcin.2013.03.013 23866184

[B78] De Luca G Parodi G Sciagrà R Venditti F Bellandi B Vergara R Migliorini A Valenti R Antoniucci D Preprocedural TIMI flow and infarct size in STEMI undergoing primary angioplasty J Thromb Thrombolysis 2013 Aug 9. [Epub ahead of print] 10.1007/s11239-013-0977-x 23928869

[B79] Stone GW Dixon SR Grines CL Cox DA Webb JG Brodie BR Griffin JJ Martin JL Fahy M Mehran R Miller TD Gibbons RJ O'Neill WW Predictors of infarct size after primary coronary angioplasty in acute myocardial infarction from pooled analysis from four contemporary trials Am J Cardiol 2007 100 1370 1375 10.1016/j.amjcard.2007.06.027 17950792

[B80] Mahaffey KW Puma JA Barbagelata NA DiCarli MF Leesar MA Browne KF Eisenberg PR Bolli R Casas AC Molina-Viamonte V Orlandi C Blevins R Gibbons RJ Califf RM Granger CB Adenosine as an adjunct to thrombolytic therapy for acute myocardial infarction: results of a multicenter, randomized, placebo-controlled trial: the Acute Myocardial Infarction Study of Adenosine (AMISTAD) trial J Am Coll Cardiol 1999 34 1711 1720 10.1016/S0735-1097(99)00418-0 10577561

[B81] Henriques JPS Zijlstra F Ottervanger JP Dambrink J-HE Hof AWJ v 't Hoorntje JCA de Boer M-J Suryapranat H Angiographic determinants of infarct size after successful percutaneous intervention for acute ST-elevation myocardial infarction: the impact of distal embolisation Neth Heart J 2002 10 353 359 PMC2499766 25696128

[B82] Bruce CJ Christian TF Schaer GL Spaccavento LJ Jolly MK O'Connor MK Gibbons RJ Determinants of infarct size after thrombolytic treatment in acute myocardial infarction Am J Cardiol 1999 83 1600 1605 10.1016/S0002-9149(99)00164-2 10392861

[B83] Christian TF Schwartz RS Gibbons RJ Determinants of infarct size in reperfusion therapy for acute myocardial infarction Circulation 1992 86 81 90 10.1161/01.CIR.86.1.81 1617793

[B84] Duconge J Miranda-Massari JR Gonzalez MJ Jackson JA Warnock W Riordan NH Pharmacokinetics of vitamin C: insights into the oral and intravenous administration of ascorbate P R Health Sci J 2008 27 7 19 18450228

[B85] Padayatty SJ Sun AY Chen Q Espey MG Drisko J Levine M Vitamin C: intravenous use by complementary and alternative medicine practitioners and adverse effects PLoS One 2010 5 e11414 10.1371/journal.pone.0011414 20628650 PMC2898816

[B86] Bjelakovic G Nikolova D Gluud LL Simonetti RG Gluud C Antioxidant supplements for prevention of mortality in healthy participants and patients with various diseases Cochrane Database Syst Rev 2012 3 CD007176 22419320 10.1002/14651858.CD007176.pub2 PMC8407395

[B87] Miller ER 3rd Pastor-Barriuso R Dalal D Riemersma RA Appel LJ Guallar E Meta-analysis: high-dosage vitamin E supplementation may increase all-cause mortality Ann Intern Med 2005 142 37 46 10.7326/0003-4819-142-1-200501040-00110 15537682

[B88] Ye Y Li J Yuan Z Effect of antioxidant vitamin supplementation on cardiovascular outcomes: a meta-analysis of randomized controlled trials PLoS One 2013 8 e56803 10.1371/journal.pone.0056803 23437244 PMC3577664

[B89] Myung SK Ju W Cho B Oh SW Park SM Koo BK Park BJ Korean Meta-Analysis Study Group Efficacy of vitamin and antioxidant supplements in prevention of cardiovascular disease: systematic review and meta-analysis of randomised controlled trials BMJ 2013 346 f10 10.1136/bmj.f10 23335472 PMC3548618

[B90] Waters DD Alderman EL Hsia J Howard BV Cobb FR Rogers WJ Ouyang P Thompson P Tardif JC Higginson L Bittner V Steffes M Gordon DJ Proschan M Younes N Verter JI Effects of hormone replacement therapy and antioxidant vitamin supplements on coronary atherosclerosis in postmenopausal women: a randomized controlled trial JAMA 2002 288 2432 2440 10.1001/jama.288.19.2432 12435256

